# Poor echocardiographic view can be a sign of the development of thoracic pneumatosis

**DOI:** 10.1186/s40981-022-00507-6

**Published:** 2022-02-27

**Authors:** Keiko Uemura, Mari Ozminsky, Chie Matsuoka, Satoki Inoue

**Affiliations:** 1 Department of Anesthesiology, Nara Medical University, 840 Shijocho, Kashihara, 634-8521 Japan; 2 Department of Anesthesiology, Fukushima Medical University, 1 Hikarigaoka, Fukushima, Fukushima 960-1295 Japan

**To the Editor**

Mechanical ventilation is an essential medical intervention for patients with severe respiratory failure. However, this intervention potentially has a risk of life-threatening adverse events, which include barotrauma or volutrauma that causes air leak into the pleural space resulting in conditions such as pneumothorax, pneumomediastinum and subcutaneous emphysema [1]. Needless to say, pneumothorax, especially tension pneumothorax, is a medical emergency while subcutaneous emphysema is not a severe adverse event. It has been suggested that subcutaneous emphysema may become apparent on chest X-rays before pneumothorax does [2]. Chest X-ray showing subcutaneous emphysema may be used as a sign of progression to thoracic pneumatosis [2]. It would be helpful if there are established signs of subcutaneous emphysema for making a diagnosis in the earlier stages. It is well known that subcutaneous emphysema can reduce the quality of ultrasound images [3]. Recently, we noticed that all patients in our previously reported cases [4–6] had had a poor echocardiographic view before developing severe thoracic pneumatosis during mechanical ventilation. We also reviewed the series of chest X-ray images of these cases before developing severe thoracic pneumatosis and found mild subcutaneous emphysema in those images (Fig. 1). Concurrently with these images, a finding of poor echocardiographic view was recorded in the medical record in each case. From these experiences, it is assumed that we could have prevented the development of life-threatening thoracic pneumatosis if we had noticed subcutaneous emphysema in the first poor echocardiographic view. Our cases suggest that we should suspect subcutaneous emphysema in cases with poor echocardiographic view during mechanical ventilation. It is likely that poor echocardiographic view can be considered as a sign of development of thoracic pneumatosis, and we should be aware of this for early prevention.

**Fig. 1:**
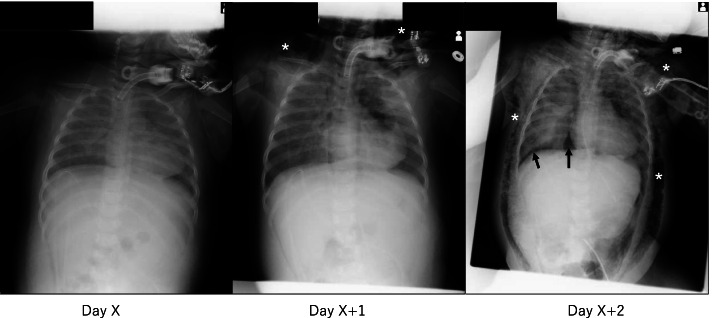
Representative X-ray images of a patient (an 8-month-old female with weight 5.7 kg and height 60 cm) [5]. On day X, good echocardiographic imaging was obtained; however, the image quality was slightly reduced than the day before. There was no remarkable change suggesting pneumatosis on the X-ray image. On day X + 1, good echocardiographic imaging was not obtained. Taking a closer look at the X-ray image, subcutaneous emphysema (asterisks) is observed around the neck. We overlooked this sign because we were distracted by the change of the X-ray image of the left lung field. On day X + 2, the patient developed severe pneumatosis. The right-side X-ray image shows extensive subcutaneous emphysema (asterisks) and pneumothorax (arrows)

## Data Availability

Not applicable.
